# Flesh Quality, Shelf Life, and Freshness Assessment of Sea Bream Reared in a Coastal Mediterranean Integrated Multi-Trophic Aquaculture System

**DOI:** 10.3390/ani15162425

**Published:** 2025-08-19

**Authors:** Simona Tarricone, Maria Antonietta Colonna, Marco Ragni, Roberta Trani, Adriana Giangrande, Grazia Basile, Loredana Stabili, Claudia Carbonara, Francesco Giannico, Caterina Longo

**Affiliations:** 1 Department of Soil, Plant and Food Sciences, University of Bari Aldo Moro, 70126 Bari, Italy; simona.tarricone@uniba.it (S.T.); marco.ragni@uniba.it (M.R.); 2 Department of Bioscience, Biotechnologies and Environment, University of Bari Aldo Moro, 70126 Bari, Italy; roberta.trani@uniba.it (R.T.); caterina.longo@uniba.it (C.L.); 3 Department of Biological and Environmental Sciences and Technologies, University of Salento, 73100 Lecce, Italy; adriana.giangrande@unisalento.it; 4 Maricoltura Mar Grande Scarl, 74122 Taranto, Italy; info@maricolturamargrande.it; 5 Institute for Water Research (IRSA)-CNR, Talassografico “A. Cerruti”, 74123 Taranto, Italy; loredana.stabili@irsa.cnr.it; 6 Department of Medical and Surgical Sciences, University of Foggia, 71122 Foggia, Italy; claudia.carbonara@unifg.it; 7 Department of Veterinary Medicine, University of Bari Aldo Moro, 70010 Valenzano, Italy

**Keywords:** sea bream, integrated multi-trophic aquaculture, flesh quality, QIM, Ionian sea

## Abstract

This study investigated the flesh quality, shelf life, and sensory freshness of sea bream (*Sparus aurata*) reared in the REMEDIA Life IMTA system, which incorporates bioremediator organisms—sponges, polychaetes, bivalves, and macroalgae—supported by artificial vertical collectors to enhance the settlement of sessile macroinvertebrates and improve environmental quality. A total of 96 fish (18 months old) were analysed, 48 farmed within the IMTA system and 48 in the conventional offshore system. Both groups received the same commercial feed. For each group, 16 fish were analysed after 1, 7, and 14 days of storage at 2 ± 1 °C to evaluate physical features, chemical and fatty acid composition, and sensory freshness. The total weight was markedly greater for fish in the IMTA group (*p* < 0.05), which showed a significantly (*p* < 0.05) longer tail. For all the storage times, the content of total saturated fatty acids was markedly higher in the control group, along with a lower concentration of polyunsaturated fatty acids (*p* < 0.05). The quality index method showed better results for the IMTA group (*p* < 0.05), particularly after 2 weeks of storage in ice. In conclusion, sea bream reared in the IMTA system showed better flesh quality, extended shelf life, and prolonged sensory freshness.

## Simple Summary

Integrated multi-trophic aquaculture (IMTA) is an innovative and sustainable farming system based on the simultaneous cultivation of different aquatic species placed on different levels of the food chain. This farming system has the advantage of reproducing natural ecosystems while using wastes produced from carnivorous fish species or shrimp as feed resources for other invertebrate organisms. We studied the effect of rearing European sea bream (*Sparus aurata*) according to an IMTA system (using sponges, bivalves, polychaetes, and seaweeds as bioremediating organisms) compared to a monocultural farming system method. Growth performances, physical and chemical characteristics, fatty acid profile and texture, lipid oxidation, and sensory properties of fillets stored for 1, 7, and 14 days were evaluated. Our results show that sea bream reared under the IMTA system have better flesh quality, prolonged shelf life, and superior sensory features compared to fish farmed in a traditional monoculture system. Through this investigation, we contribute to the growing body of evidence needed to validate IMTA as a sustainable, productive, and market-viable aquaculture strategy.

## 1. Introduction

Integrated multi-trophic aquaculture (IMTA) represents one of the most innovative and ecologically sustainable approaches to aquaculture in freshwater, brackish, and marine environments. Unlike traditional monoculture systems, IMTA involves the co-cultivation of species from different trophic levels, such as carnivores, as well as detritivorous and filtering organisms, where the waste outputs of fed species—such as finfish and shrimp—serve as inputs for extractive organisms like sponges, bivalves, polychaetes, and seaweeds [1,2]. This biologically synergistic model reduces environmental impacts such as eutrophication, sedimentation, and chemical accumulation while enhancing water quality and overall ecosystem resilience [3,4,5,6].

Extensive literature has highlighted the ecological benefits of IMTA, ranging from nutrient recycling [7,8] and water amelioration to its role in carbon sequestration [9,10] and the mitigation of ocean acidification [11]. Simultaneously, economic and social advantages are increasingly recognized. For example, consumer willingness to pay a premium for IMTA-certified products reflects growing demand for environmental friendly, welfare-conscious aquaculture [12]. However, despite its promising potential, the adoption of IMTA remains limited in many parts of Europe, particularly in the Mediterranean area, where challenges related to market development, product certification, and stakeholder acceptance persist [2].

A crucial step towards broader IMTA implementation lies in demonstrating the system’s ability to provide environmental and economic benefits along with high-quality aquaculture products [13,14,15]. To date, few studies have evaluated how IMTA practices influence the flesh quality, shelf life, and sensory attributes of target species—factors critical to consumer acceptance and marketability [16,17].

The “Maricoltura Mar Grande” (MMG) is an aquaculture facility located in Taranto (Italy), where a pilot IMTA system integrating fish with a range of bioremediator species including sponges, mussels, polychaetes, and macroalgae has been implemented since 2019 [17,18,19,20]. Several studies have been carried out in order to investigate the effects of bioremediator organisms on the environmental impact of the mariculture plant, on the quality of the water column, and on the biodiversity in the area surrounding the IMTA system [17,18,21,22].

By analysing European sea bream (*Sparus aurata*) reared within this IMTA system, we aim to provide new insights into the performance and commercial value of IMTA-raised fish. Specifically, this research evaluates key quality indicators such as texture, lipid oxidation, and freshness assessment over storage time. Through this investigation, we contribute to the growing body of evidence needed to validate IMTA as a sustainable, productive, and market-viable aquaculture strategy.

## 2. Materials and Methods

### 2.1. Rearing System

The study was carried out at the Maricoltura Mar Grande (MMG) aquaculture facility, situated along the southwestern coast of the Mar Grande in Taranto, Italy (40°25′56″ N; 17°14′19″ E), within the Northern Ionian Sea. This site hosts the REMEDIA Life integrated multi-trophic aquaculture (IMTA) system. Covering an area of 0.06 km^2^, the facility is located approximately 600 m offshore in a semi-enclosed section of the Mar Grande. It comprises fifteen floating cages (each 22 m in diameter), operating at depths ranging from 7 to 12 m, and annually produces about 100 tons of European seabass (*Dicentrarchus labrax*, Linnaeus, 1758) and gilthead sea bream (*Sparus aurata*, Linnaeus, 1758) [19]. Since 2019, half of the MMG site has been transformed into an innovative IMTA system that integrates bioremediating organisms such as sponges, polychaetes, bivalves, and macroalgae. A key innovation of this system is the deployment of artificial vertical collectors suspended in the water column. These collectors serve both as substrates for bioremediator cultivation and as sites for the natural settlement of sessile extractive invertebrates, thereby enhancing the system’s capacity to improve environmental quality and support sustainable aquaculture practices [2].

**Figure 1:**
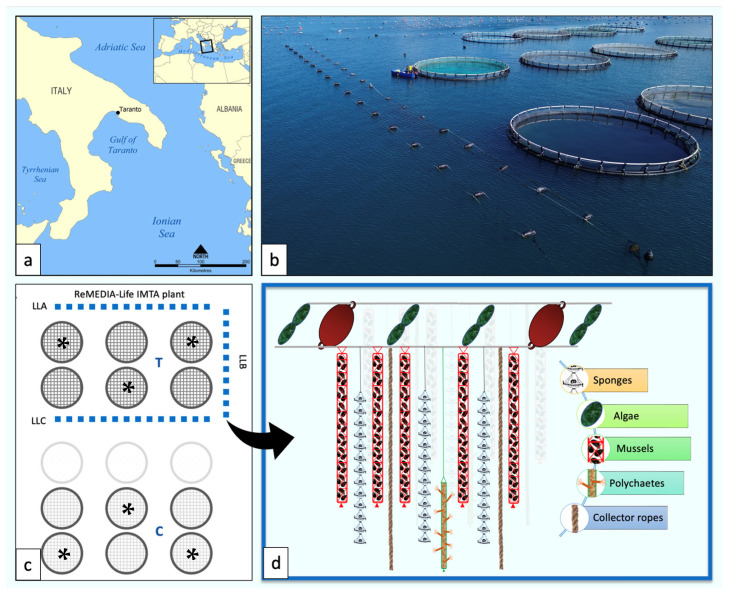
The REMEDIA Life IMTA system: (**a**) Geographic location of the IMTA system in the Northern Ionian Sea (Mar Grande of Taranto, Southern Italy); (**b**) aerial photograph of the “Maricoltura Mar Grande” facility and IMTA system; (**c**) map of the IMTA facility showing the position of the three long lines (LLA, LLB, and LLC) and the two areas: T (treatment) and C (control); (**d**) drawing of the long lines with the bioremediator organisms on the vertical collectors. The asterisk indicates the cages from which the fish were sampled for the treatment and control groups.

After an initial ex-ante monitoring phase [23,24] during which the main hydrological characteristics of the area were also measured, the MMG facility was divided into two sectors: a treatment area, equipped with bioremediators, and a control area, without them. Within the treatment sector, three long lines (LLA, LLB, and LLC) were installed in conjunction with six fish cages (Figure 1).

These long lines were outfitted with vertical collectors for invertebrate cultivation and horizontal collectors for macroalgae growth [2], and were buoy-supported to prevent sinking under biomass weight. Each long line consisted of floating buoys linked by double ropes. The space between adjacent buoys formed breeding “chambers” (for a total of 36 chambers), designed to hold bioremediator modules. These modules included vertical collectors for sponges, polychaetes, and bivalves, while macroalgae were cultivated horizontally near the surface. In addition, bare vertical collectors made of 10 m long coconut fiber ropes (2 cm wide) were installed along all long lines to encourage the natural colonization of fouling species [18,20].

The bioremediators cultured in this system included the tube worm *Sabella spallanzanii* (Gmelin, 1791), the mussel *Mytilus galloprovincialis* (Lamarck, 1819), and four sponge species *Sarcotragus spinosulus* (Schmidt, 1862), *Aplysina aerophoba* (Nardo, 1833), *Geodia cydonium* (Linnaeus, 1767), and *Hymeniacidon perlevis* (Montagu, 1814). Two macroalgae species were also cultivated: *Chaetomorpha linum* (O.F. Müller) Kützing and *Gracilaria bursa-pastoris* (S.G. Gmelin) P.C. Silva [19].

The monitoring of the main currents and environmental settings performed during the REMEDIA Life project showed that the cages chosen for the present trial (marked with asterisks in Figure 1) maintained distinct ecological features throughout the entire duration of the experiment. [22,23]. The control and IMTA cages contained fish of the same age (18 months) and size (300 ± 50 g), deriving from the same stock of juveniles, and had similar density (about 30,000 individuals/cage). Fish cultured in both groups had the same commercial feed for sea bream (RoyalMarine BREAM 4.5 mm; 4fish s.r.l., Terni, Italy), containing processed animal proteins of bird and swine origin, wheat, fish meal, soybean meal, fish oil, barley protein concentrate, corn germ oil, fish hydrolysed protein, green pea, and vitamin and mineral premix (Table 1).

Premix provides, per kg: vitamin A (6500 IU), vitamin D3 (1900 IU), vitamin E (200 mg), vitamin K3 (2500 mg), vitamin B1 (3000 mg), vitamin B2 (3000 mg), calcium pantothenate (10,000 mg), nicotinic acid (20,000 mg), vitamin B6 (2000 mg), vitamin B9 (1500 mg), cupric sulphate (5.50 mg), iron sulphate (600 mg), potassium iodide (10 mg), manganese oxide (38 mg), sodium selenite (1 mg), zinc sulphate (103 mg), calcium carbonate (15,000 mg), potassium chloride (24,100 mg), and sodium chloride (3000 mg); selenium (0.40 mg), E310 (75 mg), and E321 (150 mg).

The trial was carried out using 96 sea bream reared in the “Maricoltura Mar Grande” (MMG) aquaculture facility.

Fish were treated according to the “Council Directive 2010/63/EU on the protection of animal used for scientific purpose” [25] and to the “Ethical Justification for the Use and Treatment of Fish in Research” [26].

The growth trial lasted 18 months; at the end of the experiment, a total of 96 fish were assessed: 16 fish per each cage were analysed in triplicate for each rearing system. Fish were killed by means of hypothermia, using a mix of water and ice (1:3), according to the laws in force [27]. The fish were immediately placed in flaked ice and transported to the laboratory in refrigerated conditions. In order to reproduce the storage conditions used in fish markets, where fish are stored and sold whole and ungutted, fish were refrigerated at 2 ± 1 °C for 1, 7, and 14 days in polystyrene boxes equipped with outlets for water drainage and covered with flaked ice inside a plastic bag, as previously described by Tarricone et al. [28]. The ice/fish ratio (1:1) was kept constant throughout the storage period by re-icing the fish boxes and checking the temperature every day in order to ensure proper fish storage.

The following parameters were calculated [29,30]:

Relative Profile = 100 × (maximum height/fork length);

Cranial Index = 100 × (head length/fork length);

Condition Factor (K) = 100 × (body weight/total length^3^);

Edible Yield = 100 × (edible part weight/body weight);

Viscerosomatic Index (VSI) = 100 × (wet weight of viscera and associated fat/wet body weight);

Hepatosomatic Index (HSI) = 100 × (wet weight of liver/wet body weight) × 100.

### 2.2. Analytical Determinations

At each time of storage (1, 7, and 14 days), sixteen fish from each group were taken from their respective boxes. They were subjected to physical and chemical analyses and sensory freshness evaluation. The quality index method (QIM) and skin colour evaluations were the first to be performed. The fish were then filleted and the pH and colour analyses, texture profile analysis (TPA), and lipid oxidation analyses were performed using the left-side fillet, while the right-side ones were used to perform the Torry scheme method.

#### 2.2.1. pH, Colour, and Textural Parameters of Sea Bream Fillets

The pH values were measured using a portable instrument (Model HI 9025) with an electrode (FC 230C; both from Hanna Instruments, Villafranca Padovana, PD, Italy) and by performing a two-point calibration (at pH equal to 7.01 and 4.01).

The colorimetric features (L* = lightness, a* = redness, b* = yellowness) of the fish fillets were determined using a Hunter Lab Miniscan™ XE Spectrophotometer (Model 4500/L, 45/0 LAV, 3.20 cm diameter aperture, 10° standard observer focusing at 25 mm, illuminant D65/10; Hunter Associates Laboratory Inc., Reston, VA, USA) by taking three readings for each sample along the left-side fillet (in correspondence with the cranial, middle and caudal fin regions). The instrument was normalized to a standard white tile provided with the instrument before performing the analysis [31].

The rheological properties of the raw fish fillets were assessed using an Instron 5544 Universal Testing Machine (Instron Corp., Norwood, MA, USA). Texture profile analysis (TPA) was performed using a flat steel probe with a 25 mm diameter, through a double compression test elaborated by the incorporated software. From the left side of each fish, three samples with a square surface (2 × 2 cm) and a height of 0.5 cm were excised from three different areas along the fillet (as described for colour assessment). The mean values of the measurements for each test per fish were retained for statistical analysis. The fillet samples underwent two compression cycles, 2 min apart, at a speed rate of 0.6 m/s. A compression equal to 75% of the initial height of the sample was performed for both cycles. The following parameters were determined: hardness (N/cm^2^), expressed as the maximum force required to compress the sample; cohesion force resilience, that is, the extent to which the sample could be deformed prior to rupture (A2/A1, where A1 and A2 are the total energy required for the first and second compression, respectively); springiness (cm), i.e., the ability of the sample to recover its original form after the deforming force is removed; gumminess (N/cm^2^), that is, the force needed to disintegrate a semisolid sample to a steady state of swallowing (hardness × cohesiveness); and chewiness (N/cm), i.e., the work needed to chew a solid sample to a steady state of swallowing (springiness × gumminess) [32,33].

#### 2.2.2. Chemical and Fatty Acid Analysis and Lipid Oxidation of Sea Bream Fillets

Right-side fillets were rapidly chopped, combined in a pool, and homogenized for 1 min. The procedures described by the Association of Official Agricultural Chemistry (AOAC) were used to assess moisture, ether extract, raw protein, and the ash content [34]. The total lipids were extracted using a 2:1 chloroform/methanol (*v*/*v*) solution to determine the fatty acid profile [35]. The fatty acids were then methylated using a KOH/methanol 2N solution [36] and analyzed by gas chromatography (Shimadzu GC-17A—Shimadzu Italia, Milan, Italy) using a silicone–glass capillary column (70% Cyanopropyl Polysilphenylenesiloxane BPX 70 by Thermo Scientific—Thermo Fisher Scientific, Rodano, MI, Italy—length = 60 m, internal diameter = 0.25 mm, film thickness = 0.25 μm). The starting temperature was 135 °C for 7 min, which then increased by 4 °C/min up to 210 °C. Fatty acids were identified by comparing retention times to authentic standards for percentage area normalization. Fatty acids were expressed as percentage (*wt*/*wt*) of total methylated fatty acids.

The food risk factors of meat were determined by calculating the atherogenic (AI) and thrombogenic (TI) indices [37]:

AI = [(C12:0 + 4 × C14:0 + C16:0)] ÷ [ΣMUFA + Σn-6 + Σn-3];

TI = [(C14:0 + C16:0 + C18:0)] ÷ [(0.5 × ΣMUFA + 0.5 × Σn-6 + 3 × Σn-3 + Σn-3)/Σn-6];

where MUFA are monounsaturated fatty acids.

Lipid oxidation was evaluated on fillets stored at 2 ± 1 °C for 1, 7, and 14 days by measuring the concentration of 2-thiobarbituric acid reactive substances (T-BARS) and expressed as mg malondialdehyde (MDA)/kg meat [38].

#### 2.2.3. Freshness Assessment of Sea Bream by QIM and Torry Scheme

Sensory freshness analysis was performed using the QI method and the Torry scheme, which are the most common procedures used for evaluating the freshness of raw and cooked fish, respectively [39]. Sensory analyses were performed by eight panelists recruited from the Department of Soil, Plant and Food Sciences of the University of Bari Aldo Moro. These panelists were selected for their expertise in the descriptive analysis of fish freshness parameters and for their experience with fish quality evaluation. Before the main evaluation, training sessions with sea bass fillets were conducted to train the panelists on how to use the QIM and Torry schemes developed for the analysis of snakehead fish fillets [40].

The QI method is based on the evaluation of five attributes: skin, including lightness (0–2), odour (0–3), and texture (0–2); eye characteristics, including colour (0–2) and conformation (0–2); gills, with regards to colour (0–2), mucus (0–2), and odour (0–3); colour of flesh fillet (0–2); and viscera solution (0–2). For each of the above-described attributes, the panelists gave each item a score ranging within the interval indicated and the sum of scores obtained for all the items was recorded. Finally, QI ranged from 0 to 22, with lower scores reflecting higher quality of fish.

For the analysis of the odour and flavour of the cooked sea bream fillets, six slices (2 × 6 cm) were cut from the right-side fillets, wrapped in aluminium foil paper, placed in a perforated stainless-steel pan, and steam cooked for 10 min at 95–100 °C. After cooking, the samples were blind coded with a 3-digit random number and served to the panelists for evaluation using the Torry scheme method developed by Shewan et al. [41], with some modifications made by Martinsdottir et al. [39] for medium-fatty fish. The Torry scheme evaluation is a descriptive 10-point scale with higher scores reflecting premium quality. An average score of ≤5.5 was used as the sensory rejection point.

### 2.3. Statistical Analysis

Statistical analyses were performed using Python software (3.11.13) [42]. Flesh quality traits were analyzed by ANOVA for repeated measures, with the rearing system as a non-repeated factor and storage time and their interaction as repeated factors. Results are reported as means and standard errors of the means (SEM); significant effects were declared at *p* < 0.05. When significant, means were compared using Student’s *t*-test.

## 3. Results

### 3.1. Growth Parameters

The growth performances of sea bream reared in different conditions are shown in Table 2. The IMTA system significantly increased (*p* < 0.05) the total body weight of sea bream and their tail length, while the total body length was unaffected by the rearing system.

Sea bream from the IMTA group showed a markedly greater cranial index (*p* < 0.05) as compared to the control group. The edible yield was significantly (*p* < 0.05) higher in the control group, which resulted in a greater hepatosomatic index (*p* < 0.05).

#### 3.1.1. pH, Colour, and Textural Parameters and Lipid Oxidation of Sea Bream Fillets

The results concerning the pH, physical parameters, and lipid oxidation of sea bream fillets are shown in Table 3. The pH values of the fillet are not affected by the rearing system or by the storage time.

As for the dorsal skin area, the rearing conditions did not influence the colour indices, while the storage time significantly (*p* < 0.01) decreased the L* value from day 1 to day 14 and increased the b* index at the end of the storage period as compared to both the previous sampling days in both groups. The a* values were greater (*p* < 0.01) at 14 days of storage in comparison with day 1, both for the control and IMTA groups.

In both groups, the colour indices of the fillets were influenced by the storage time: the L and b* values significantly (*p* < 0.01) increased over time, while the a* values decreased (*p* < 0.01) from day 1 to day 14.

As for the textural parameters, hardness, gumminess, cohesion force, and chewiness decreased significantly (*p* < 0.01) from day 1 to day 14 of storage for both groups, and the interaction between the rearing conditions and the storage was significant (*p* < 0.05).

The IMTA rearing system led to a significantly (*p* < 0.01) lower concentration of MDA in the fillet as compared to the control group on days 1 and 14 of storage. The storage time determined a significant increase (*p* < 0.01) in the MDA concentration at day 14 with respect to day 1 and day 7 in both groups, and also their interaction was significant (*p* < 0.01).

#### 3.1.2. Chemical and Fatty Acid Analysis of Sea Bream Fillets

The chemical composition of sea bream fillets is shown in Table 4. At day 7, fillets of fish from IMTA system showed a greater percentage of moisture with respect to the control group. Storage time significantly lowered (*p* < 0.05) the moisture percentage from day 1 to day 14; also, the interaction between the rearing conditions and the storage time was significant (*p* < 0.05). No significant effects of the IMTA system and the storage time were observed for the other parameters analyzed.

Table 5 shows the fatty acid profile of the sea bream fillets. Fillets from the IMTA system contained a lower concentration of myristic (*p* < 0.01), palmitic (*p* < 0.01), and total saturated fatty acids (*p* < 0.05) as compared to the control groups for all sampling times. The concentrations of C20:0 and C22:0 in fillets of fish from the IMTA system were significantly higher as compared to the control group (*p* < 0.05) at all storage times. At days 1 and 14, fillets of the IMTA group showed a higher value of C23:0 (*p* < 0.01).

Storage time from day 1 to days 7 and 14 significantly increased the fillet content of palmitic and stearic acids (*p* < 0.05) and the concentration of total SFA (*p* < 0.01). In both groups, the storage of fish from day 1 to day 14 significantly increased the concentrations of C22:0 (*p* < 0.05) and C23:0 (*p* < 0.01).

The IMTA system led to a lower (*p* < 0.05) concentration of oleic acid in the sea bream fillets; conversely, in the control group, the storage of the fish from day 1 to day 14 significantly decreased (*p* < 0.05) the oleic acid concentration.

The concentration of γ–linolenic acid was markedly greater (*p* < 0.05) in the control group as compared to the IMTA system only on day 1 of analysis. The control system determined a progressive decrease in the concentration of this fatty acid from day 1 to days 7 and 14 (*p* < 0.05). The IMTA system led to a higher concentration of the C20:3n-6 fatty acid for all the three storage times (*p* < 0.05). For both groups, the concentration of C20:3n-6 markedly lowered from day 1 to days 7 and 14 (*p* < 0.05). The concentration of arachidonic acid was unaffected by the rearing system, while it showed a significant decrease (*p* < 0.05) from day 1 to days 7 and 14 in both groups. The concentration of the long-chain fatty acids DPA and DHA was markedly greater (*p* < 0.05) in the IMTA group regardless of the day of storage.

The total PUFA concentration was significantly higher in the IMTA group as compared to the control for all the storage times (*p* < 0.05). In both groups, these fatty acids showed a significant decrease from day 1 to days 7 and 14. The concentration of total n-6 was unaffected by the rearing system, while it significantly decreased (*p* < 0.05) from day 1 to days 7 and 14 in both groups. The IMTA system led to a significantly greater value (*p* < 0.05) of total n-3 concentration over time; fillets from the control group showed a marked decrease (*p* < 0.05) in concentration of total n-3 from day 1 to day 14 of storage. Atherogenic and thrombogenic indices were markedly higher (*p* < 0.05) in the fillets from the control groups for all the sampling times; the AI significantly increased (*p* < 0.05) from day 1 to day 14 of storage in the control group, while the TI increased (*p* < 0.05) from day 1 to days 7 and 14 in both groups.

#### 3.1.3. Sensorial Analysis of Sea Bream Fillets

**Figure 2:**
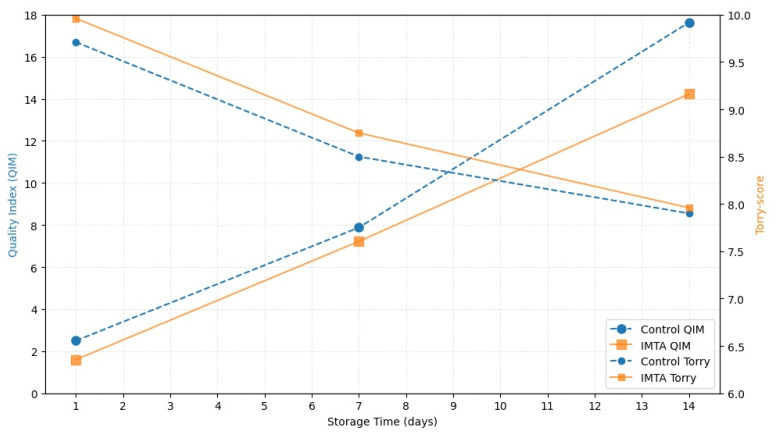
Changes in quality index and eating quality (Torry score) of sea bream during storage.

The results of the freshness assessment of sea bream fillets are shown in Table S1 and Figure 2. The IMTA system provided markedly (*p* < 0.05) better scores for fish quality at days 1 and 14 of storage as compared to the control system. For both rearing systems, the QIM scores recorded at day 14 were significantly higher (*p* < 0.01) as compared to day 1, showing the evolution of the visual and odour judgement of the fish. The rearing system did not affect the Torry score of cooked fillets. This method highlighted the progressive decrease over time of the score attributed to cooked fillets, with significant (*p* < 0.01) differences between days 1 and 14 for both rearing systems, confirming the trend observed for fresh fish.

Figure 2 shows the synergistic judgement scores from the QI and Torry scheme methods. The intersection point extrapolated from the lines of the two methods, for each rearing system, shows the storage time until which the fish preserves its optimal quality, as the result of global evaluation of visual appearance, odour, and fillet texture after cooking.

For both rearing systems, the end point values registered on day 14, although significantly lower in comparison with day 1, fall within the range of fish quality acceptability. For the IMTA rearing system, the intersection point between the QI and Torry methods was found at day 10, that is, one day later as compared to the control group, evidencing the effectiveness of the IMTA method in prolonging and improving overall fish quality.

## 4. Discussion

Integrated multi-trophic aquaculture has been developed as an innovative and sustainable farming system thanks to the synergic cultivation of several aquatic species across different levels of the food chain. This system has the advantage of improving the quality of the environment, thus enhancing microbial community composition and dynamics [21]. Therefore, the improved quality of the environment is supposed to positively affect the quality of the farmed fish. Indeed, although limited research has been carried out on IMTA, previous experiments conducted on the most important market fish species, such as the European sea bass and the gilthead sea bream, have given promising results [5].

The IMTA rearing system determined a greater body weight, tail length, and cranial index as compared to the control group. The greater body weight may be the result of the better trophic resources available, which lead to enhanced weight gain, as previously found by other authors [43,44]. The longer tail recorded for IMTA fish shows lower fin erosion in this group. Alterations of fin length and shape may be caused by various factors, among which are the aggressive behavior between fish, nutritional deficiencies, and bacterial infection [45]. Fin erosion is an important parameter in aquaculture because of the issues it creates for fish aesthetic and survival [46].

In other studies, it has been reported that sea bream from the IMTA system showed better physical features with regards to the colour of the skin and flesh, while in our study no differences were observed for the colour parameters. Flesh quality is the result of the complex relationship between fish, feeding, and rearing conditions, which affect the physical–chemical parameters, the post mortem degradation processes and, finally, the eating quality of fish [47,48]. Many changes in fish fillets occur during storage, including in texture, which is considered an important quality marker for fish palatability [49]. The fillet texture usually shows the highest values during the first days of storage, with a progressive decrease from day 7 onwards. In this study, the texture parameters were best until day 7, while storage for 14 days showed a decrease in quality. These results are in general agreement with previous findings, which reported that fillet softness during storage may be due to the combination of enzymatic autolysis and microbial spoilage [49,50,51]. Fish spoilage bacterial activity is influenced by an increase in pH during storage due to the accumulation of alkaline compounds. In this study, in accordance with other authors [52], we found an increase in the pH of fillets during storage, but this parameter was unaffected by the fish rearing system.

The more balanced environment achieved by IMTA improves water quality and the ecosystem, with positive effects on fish health, disease resistance, and oxidative status [5]. In this study, we found a lower concentration of MDA in IMTA-reared fish, showing the positive influence of this rearing system on the oxidative stability of the fillet.

Seafood is a unique source of long-chain fatty acids, including EPA, DHA and DPA, which play a crucial role in preventing cardiovascular diseases, inflammatory processes, and cancer, as extensively reviewed by various authors [6,53,54,55]. The IMTA system has been reported to provide nutritional benefits for farmed fish, particularly with regard to higher levels of fatty acids of the n-3 series. Indeed, in our study we found a better fatty acid profile in fish from the IMTA system, as shown by higher concentrations of DPA, DHA, PUFA, and total n-3 fatty acids, along with lower values for the AI and TI indices. This result provides great advantages for human health, thus encouraging the application of IMTA compared to conventional monoculture systems [56].

So far, sensory analysis is the most important method for freshness evaluation, though its limitation lies in the training of panels able to perform sensory evaluation by objective criteria [57]. In this study, the assessment of fish freshness, as analyzed by the QI method and Torry scheme, highlighted better outcomes for the IMTA system. In particular, the QI method evidenced a better odour profile, with pronounced notes of marine and iodine markers of freshness, probably due to the influence of the algae and filtering species present in the IMTA system [5,58]. The intersection of the lines referring to the QI and Torry methods showed prolonged fish quality for the IMTA rearing system as compared to the control group. Furthermore, the linear relationship of the QI method performed on whole raw fish may be used to predict the Torry scheme, thus replacing the sensory evaluation of cooked samples [39,57]. Alasalvar et al. [59] found that the limit of acceptability for cultured sea bream stored in ice was about 17–18 days, during which the odour of sea bream decreased from 10, corresponding to strong seaweed odour, to 3, perceived as putrid or spoiled; they also reported the presence of a fresh smell for up to ten days, as also found in this study. Moreover, the authors found that cooking may mask undesirable changes by removing volatile odours during cooking. Therefore, the combined use of the two freshness assessment methods may provide a better and more objective evaluation [60,61].

Finally, the IMTA system seems to exert effects on the microbiological status of the gut, as highlighted by ongoing research on the microbiome (personal communication).

## 5. Conclusions and Future Direction

Sea bream reared under the IMTA system exhibited improved flesh quality, extended shelf life, and prolonged freshness characteristics compared to fish farmed in a traditional monoculture system. These findings highlight the potential of IMTA as a sustainable aquaculture strategy capable of enhancing productivity while reducing environmental impact. In the future, the advancement of fish farming will rely increasingly on techniques that optimize production in harmony with ecological preservation. In this context, the IMTA approach is expected to attract growing interest for other species as well, thanks to its proven environmental benefits and the high quality of the fish produced, as well as to its ability to crease social and economic value.

## Figures and Tables

**Table 1:** Chemical composition and fatty acid profile of the diet.

	%
Chemical composition (% on DM basis)	
Moisture	6.20
Crude Protein	44.58
Total Lipid	16.05
Total N-free extracts	13.03
Fiber	1.90
Ash	9.13
Gross Energy (MJ/kg)	20.21
Fatty Acid Profile (% FA methyl esters)	
C14:0	5.3
C15:0	0.45
C16:0	15.7
C17:0	5.2
C18:0	3.6
C20:0	1.7
C16:1 n-7	7.6
C162 n-4	1.0
C16:3 n-4	1.0
C18:1 n-9	13.7
C18:1 n-7	4.5
C20:1 n-9	3.3
C18:2 n-6	5.0
C20:2 n-6	0.5
C18:3 n-6	1.1
C18:3 n-3	1.8
C18:4 n-3	1.6
C20:4 n-3	0.7
C20:5 n-3	11.2
C22:5 n-3	0.85
C22:6 n-3	11.3

**Table 2:** Growth performances of sea bream from the different rearing systems.

	Control	IMTA	SEM ^1^	*p*-Value
Total body weight (g)	277.59 ^b^	331.44 ^a^	0.863	0.046
Total body length (cm)	26.74	26.25	0.273	0.082
Fork length (cm)	24.95	24.46	0.277	0.091
Tail length (cm)	5.24 ^b^	6.03 ^a^	0.074	0.027
Relative profile (%)	36.45	36.19	0.146	0.857
Cranial index (%)	20.99 ^B^	24.56 ^A^	0.863	0.001
Condition factor (%)	1.45	1.83	0.032	0.145
Edible yield (%)	38.15 ^A^	32.70 ^B^	0.155	0.001
Viscerosomatic Index (%)	6.01	5.19	0.107	0.451
Hepatosomatic index (%)	1.13 ^a^	0.96 ^b^	0.081	0.015

^1^ Standard Error of Means. ^A,B^: *p* < 0.01; ^a,b^: *p* < 0.05.

**Table 3:** pH and colour indices performed during ice storage on the dorsal skin area and on the fillets of sea bream from different rearing systems.

Parameters	Control			IMTA			SEM ^1^	Effects ^2^		
	1	7	14	1	7	14		R	S	R × S
pH	6.26	6.33	6.34	5.96	6.08	6.18	0.079	0.076	0.156	0.159
Colour indices of sea bream dorsal skin area										
L*	59.09 ^X^	52.49 ^XY^	49.16 ^Y^	48.39 ^X^	46.03 ^XY^	43.09 ^Y^	0.985	0.231	0.006	0.061
a*	−2.14 ^Y^	−2.40 ^XY^	−2.72 ^X^	−1.90 ^Y^	−2.12 ^XY^	−2.40 ^X^	0.209	0.084	0.002	0.074
b*	4.15 ^Y^	4.49 ^Y^	5.52 ^X^	4.14 ^Y^	4.21 ^Y^	4.98 ^X^	0.319	0.091	0.001	0.043
Colour indices of sea bream fillet										
L*	59.33 ^Y^	62.03 ^XY^	65.08 ^X^	58.14 ^Y^	60.38 ^XY^	62.09 ^X^	0.985	0.231	0.006	0.081
a*	−1.14 ^Y^	−1.40 ^XY^	−1.52 ^X^	−1.09 ^Y^	−1.23 ^XY^	−1.38 ^Y^	0.209	0.084	0.002	0.054
b*	−0.15 ^Y^	−0.09 ^XY^	0.20 ^X^	−0.24 ^Y^	−0.19 ^XY^	0.58 ^X^	0.319	0.091	0.001	0.073
Hardness (N)	12.36 ^X^	10.10 ^XY^	6.38 ^Y^	12.90 ^X^	9.98 ^XY^	9.12 ^Y^	0.937	0.353	0.001	0.026
Gumminess (N)	5.78 ^X^	4.45 ^XY^	3.25 ^Y^	5.55 ^X^	4.18 ^XY^	2.64 ^Y^	0.232	0.294	0.001	0.034
Springiness (mm)	2.36	1.94	1.64	2.29	1.61	1.35	0.064	0.077	0.091	0.121
Cohesion Force Resilience	0.79 ^X^	0.54 ^XY^	0.49 ^Y^	0.76 ^X^	0.60 ^XY^	0.57 ^Y^	0.064	0.072	0.008	0.031
Chewiness (N × mm)	13.56 ^X^	11.11 ^XY^	8.56 ^Y^	12.68 ^X^	11.67 ^XY^	8.57 ^Y^	0.557	0.221	0.001	0.021
MDA (mg/kg)	0.18 ^aY^	0.22 ^Y^	0.50 ^aX^	0.12 ^bY^	0.18 ^Y^	0.32 ^bX^	0.018	0.043	0.002	0.001

^1^ Standard error of means; ^2^ effects: R, rearing system; S, storage time; R × S, interaction. Differences between rearing systems: ^a;b^: *p* < 0.05; differences between the storage periods: ^X,Y^: *p* < 0.01.

**Table 4:** Chemical composition and lipid oxidation of fillets of sea bream from the different rearing systems during storage.

Parameters	Control			IMTA			SEM ^1^	Effects ^2^		
	1	7	14	1	7	14		R	S	R × S
Moisture	72.59 ^x^	71.23 ^bxy^	70.11 ^y^	72.86 ^x^	72.31 ^ax^	70.08 ^y^	0.340	0.017	0.025	0.010
Protein	19.38	19.50	19.72	19.20	19.27	19.88	0.284	0.155	0.156	0.077
Lipid	5.61	5.99	6.35	5.50	5.81	6.36	0.264	0.082	0.096	0.101
Ash	1.34	1.72	1.82	1.34	1.65	1.97	0.121	0.065	0.071	0.059
N-free extract	1.08	1.52	1.70	1.10	1.41	1.71	0.208	0.743	0.124	0.069

^1^ Standard error of means; ^2^ effects: R, rearing system; S, storage time; R × S, interaction. Differences between rearing systems: ^a;b^: *p* < 0.05; differences between the storage periods: ^x,y^: *p* < 0.05.

**Table 5:** Fatty acid profiles of fillets of sea bream from the different rearing systems during storage.

Parameters	Control			IMTA			SEM ^1^	Effects ^2^		
	1	7	14	1	7	14		R	S	R × S
C10:0	0.02	0.03	0.03	0.04	0.04	0.05	0.002	0.236	0.804	0.166
C12:0	0.07	0.07	0.08	0.05	0.06	0.07	0.002	0.229	0.062	0.113
C14:0 (Myristic)	2.45 ^A^	2.51 ^A^	2.58 ^A^	1.91 ^B^	2.11 ^B^	2.16 ^B^	0.047	0.002	0.372	0.032
C15:0 (Pentadecylic)	0.27	0.29	0.31	0.24	0.25	0.28	0.005	0.361	0.060	0.372
C16:0 (Palmitic)	14.88 ^Ay^	16.16 ^Ax^	17.96 ^Ax^	13.72 ^By^	14.52 ^Bx^	14.89 ^Bx^	0.093	0.003	0.013	0.043
C17:0	0.24	0.24	0.26	0.25	0.29	0.30	0.009	0.144	0.062	0.086
C18:0 (Stearic)	4.12 ^y^	4.28 ^x^	4.64 ^x^	3.81 ^y^	4.24 ^x^	4.28 ^x^	0.054	0.337	0.041	0.117
C20:0	0.12 ^b^	0.13 ^b^	0.16 ^b^	0.43 ^a^	0.44 ^a^	0.47 ^a^	0.023	0.023	0.061	0.084
C21:0	0.57	0.68	0.68	0.59	0.68	0.69	0.024	0.808	0.149	0.561
C22:0	0.19 ^by^	0.22 ^bxy^	0.25 ^bx^	0.29 ^ay^	0.32 ^axy^	0.35 ^ax^	0.009	0.036	0.027	0.014
C23:0	1.21 ^BY^	1.52 ^XY^	1.78 ^AY^	1.46 ^AY^	1.58 ^XY^	1.74 ^AX^	0.037	0.008	0.006	0.016
Total SFA ^3^	24.19 ^aY^	26.07 ^aX^	28.78 ^aX^	22.81 ^bY^	24.57 ^bX^	25.11 ^bX^	0.109	0.018	0.001	0.022
C16:1 n-9s	4.16 ^a^	3.69	3.66 ^a^	3.51 ^b^	3.68	3.41 ^b^	0.053	0.035	0.320	0.088
C17:1	0.16	0.14	0.14	0.19	0.17	0.17	0.004	0.074	0.063	0.083
C18:l n-9 (Oleic)	34.12 ^bx^	33.96 ^bxy^	32.58 ^by^	35.37 ^a^	34.41 ^a^	34.34 ^a^	0.188	0.032	0.045	0.057
C20:l n-9 (Eicosanoic)	0.36	0.34	0.29	0.33	0.32	0.30	0.006	0.155	0.195	0.411
C24:1 n-9	0.53	0.50	0.48	0.48	0.48	0.47	0.009	0.190	0.529	0.394
Total MUFA ^4^	39.41	38.71	37.22	39.96	39.14	38.76	0.220	0.097	0.057	0.067
C18:2 n6 (linoleic)	21.92	21.70	21.52	22.74	22.18	22.43	0.166	0.735	0.151	0.754
C18:3 n-3 (α-linolenic)	1.94	1.73	1.62	1.67	1.75	1.75	0.072	0.071	0.056	0.131
C18:3 n-6 (γ -linolenic)	4.24 ^a,x^	3.56 ^y^	3.33 ^y^	3.57 ^b^	3.53	3.48	0.039	0.038	0.043	0.148
C20:3 n-6	0.34 ^bx^	0.27 ^by^	0.25 ^by^	0.41 ^ax^	0.37 ^ay^	0.34 ^ay^	0.025	0.018	0.047	0.053
C20:3 n-3	0.20	0.18	0.17	0.18	0.18	0.16	0.004	0.202	0.055	0.486
C20:4 n-6 ARA	0.41 ^x^	0.35 ^y^	0.34 ^y^	0.45 ^x^	0.38 ^y^	0.35 ^y^	0.029	0.075	0.032	0.082
C20:5 n-3 (eicosapentaenoic, EPA)	0.35	0.33	0.33	0.32	0.32	0.32	0.009	0.077	0.069	0.066
C22:5 n-3 (docosapentaenoic, DPA)	1.32 ^b^	1.32 ^b^	1.31 ^b^	1.53 ^a^	1.50 ^a^	1.42 ^a^	0.030	0.023	0.072	0.334
C22:6 n-3 (docosahexaenoic, DHA)	4.81 ^b^	4.62 ^b^	4.39 ^b^	5.47 ^a^	5.30 ^a^	5.17 ^a^	0.152	0.043	0.061	0.074
Total PUFA ^5^	35.71 ^bx^	34.23 ^by^	33.39 ^by^	37.00 ^ax^	35.63 ^ay^	35.54 ^ay^	0.255	0.039	0.047	0.048
Total n-6 ^6^	27.20 ^x^	26.10 ^y^	25.61 ^y^	27.81 ^x^	26.96 ^y^	26.77 ^y^	0.142	0.073	0.041	0.098
Total n-3 ^7^	8.62 ^bx^	8.17 ^bxy^	7.83 ^by^	9.25 ^a^	9.09 ^a^	8.92 ^a^	0.084	0.041	0.037	0.038
n-3/n-6	0.32	0.31	0.31	0.33	0.34	0.33	0.003	0.069	0.081	0.132
AI (Atherogenic Index)	0.33 ^ay^	0.36 ^axy^	0.40 ^ax^	0.28 ^b^	0.31 ^b^	0.32 ^b^	0.094	0.039	0.042	0.084
TI (Thrombogenic Index)	0.36 ^ay^	0.42 ^ax^	0.45 ^ax^	0.29 ^by^	0.35 ^bx^	0.36 ^bx^	0.074	0.027	0.034	0.033

^1^: Standard error of means. ^2^ Effects: R, rearing system; S, storage time; R x S, interaction. ^3^ Total SFA—saturated fatty acids (sum of C10:0 + C12:0 + C14:0 + C15:0 + C16:0 + C17:0 + C18:0 + C20:0 + C21:0 + C22:0 + C23:0); ^4^ Total MUFA—monounsaturated fatty acids (sum of C16:1 n-9 + C17:1 + C18:1 n-9 + C20:1 n-9 + C24:1 n-9); ^5^ Total PUFA—polyunsaturated fatty acids (sum of n-6 + n-3); ^6^ Total n-6 (sum of C18:2 n-6 + C18:3 n-6 + C20:3 n-6 + C20:4 n-6); ^7^ Total n-3 (sum of C18:3 n-3+ C20:3 n-3 + C20:5 n-3 + C22:5 n-3 + C22:6 n-3). Differences between rearing systems: ^A;B^: *p* < 0.01, ^a;b^: *p* < 0.05; differences between the storage periods: ^X,Y^: *p* < 0.01, ^x,y^: *p* < 0.05.

## Data Availability

Data are contained within the article.

## Supplementary Materials

The following supporting information can be downloaded at https://www.mdpi.com/article/10.3390/ani15162425/s1. Table S1. Freshness assessment of sea bream fillets.

## References

[1] Chopin F., Schulmann K., Skrzypek E., Lehmann J., Dujardin J.R., Martelat J.E., Lexa O., Corsini M., Edel J.B., Štípská P. (2012). Crustal Influx, Indentation, Ductile Thinning and Gravity Redistribution in a Continental Wedge: Building a Moldanubian Mantled Gneiss Dome with Underthrust Saxothuringian Material (European Variscan Belt). Tectonics.

[2] Reid G.K., Lefebvre S., Filgueira R., Robinson S.M.C., Broch O.J., Dumas A., Chopin T.B.R. (2020). Performance Measures and Models for Open-water Integrated Multi-trophic Aquaculture. Rev. Aquac..

[3] Azhar M., Memiş D. (2023). Application of the IMTA (Integrated Multi-Trophic Aquaculture) System in Freshwater, Brackish and Marine Aquaculture. Aquat. Sci. Eng..

[4] Khanjani M.H., Zahedi S., Mohammadi A. (2022). Integrated Multitrophic Aquaculture (IMTA) as an Environmentally Friendly System for Sustainable Aquaculture: Functionality, Species, and Application of Biofloc Technology (BFT). Environ. Sci. Pollut. Res..

[5] Rosati S., Maiuro L., Lombardi S.J., Iaffaldano N., Di Iorio M., Cariglia M., Lopez F., Cofelice M., Tremonte P., Sorrentino E. (2025). Integrated Biotechnological Strategies for the Sustainability and Quality of Mediterranean Sea Bass (*Dicentrarchus labrax*) and Sea Bream (*Sparus aurata*). Foods.

[6] Turlybek N., Nurbekova Z., Mukhamejanova A., Baimurzina B., Kulatayeva M., Aubakirova K.M., Alikulov Z. (2025). Sustainable Aquaculture Systems and Their Impact on Fish Nutritional Quality. Fishes.

[7] Toledo-Guedes K., Atalah J., Izquierdo-Gomez D., Fernandez-Jover D., Uglem I., Sanchez-Jerez P., Arechavala-Lopez P., Dempster T. (2024). Domesticating the Wild through Escapees of Two Iconic Mediterranean Farmed Fish Species. Sci. Rep..

[8] Batır E., Metin Ö., Yıldız M., Özel O.T., Fidan D. (2024). Sustainable Land-Based IMTA: Holistic Management of Finfish, Mussel, and Macroalgae Interactions, Emphasizing Water Quality and Nutrient Dynamics. J. Environ. Manag..

[9] Mhalhel K., Levanti M., Abbate F., Laurà R., Guerrera M.C., Aragona M., Porcino C., Briglia M., Germanà A., Montalbano G. (2023). Review on Gilthead Seabream (*Sparus aurata*) Aquaculture: Life Cycle, Growth, Aquaculture Practices and Challenges. J. Mar. Sci. Eng..

[10] Marhuenda-Egea F.C., Sánchez-Jerez P. (2025). Metabolomic Insights into Wild and Farmed Gilthead Seabream (*Sparus aurata):* Lipid Composition, Freshness Indicators, and Environmental Adaptations. Molecules.

[11] Rusco G., Roncarati A., Di Iorio M., Cariglia M., Longo C., Iaffaldano N. (2024). Can IMTA System Improve the Productivity and Quality Traits of Aquatic Organisms Produced at Different Trophic Levels? The Benefits of IMTA—Not Only for the Ecosystem. Biology.

[12] Piper L., De Cosmo L.M., Sestino A., Giangrande A., Stabili L., Longo C., Guido G. (2021). Perceived Social Welfare as a Driver of Green Products Consumption: Evidences from an Integrated Multi-Trophic Aquaculture Production. Curr. Res. Environ. Sustain..

[13] Cangiano T., DellaGreca M., Fiorentino A., Isidori M., Monaco P., Zarrelli A. (2001). Lactone Diterpenes from the Aquatic Plant Potamogeton Natans. Phytochemistry.

[14] Cangiano T., Dellagreca M., Fiorentino A., Isidori M., Monaco P., Zarrelli A. (2002). Effect of Ent-Labdane Diterpenes from Potamogetonaceae on Selenastrum Capricornutum and Other Aquatic Organisms. J. Chem. Ecol..

[15] Burić M., Bavčević L., Grgurić S., Vresnik F., Križan J., Antonić O. (2020). Modelling the Environmental Footprint of Sea Bream Cage Aquaculture in Relation to Spatial Stocking Design. J. Environ. Manag..

[16] Shpigel M., Ari T.B., Shauli L., Odintsov V., Ben-Ezra D. (2016). Nutrient Recovery and Sludge Management in Seabream and Grey Mullet Co-Culture in Integrated Multi-Trophic Aquaculture (IMTA). Aquaculture.

[17] Ferreira J.G., Saurel C., Ferreira J.M. (2012). Cultivation of Gilthead Bream in Monoculture and Integrated Multi-Trophic Aquaculture. Analysis of Production and Environmental Effects by Means of the FARM Model. Aquaculture.

[18] Trani R., Pierri C., Schiavo A., Lazic T., Mercurio M., Coccia I., Giangrande A., Longo C. (2025). Response of Hard-Bottom Macro-Zoobenthos to the Transition of a Mediterranean Mariculture Fish Plant (Mar Grande of Taranto, Ionian Sea) into an Integrated Multi-Trophic Aquaculture (IMTA) System. J. Mar. Sci. Eng..

[19] Giangrande A., Pierri C., Arduini D., Borghese J., Licciano M., Trani R., Corriero G., Basile G., Cecere E., Petrocelli A. (2020). An Innovative IMTA System: Polychaetes, Sponges and Macroalgae Co-Cultured in a Southern Italian In-Shore Mariculture Plant (Ionian Sea). J. Mar. Sci. Eng..

[20] Aguilo-Arce J., Ferriol P., Puthod P., Trani R., Longo C. (2024). The remedia life integrated multitrophic aquaculture system as a powerful sponge biomass supply. Biol. Mar. Mediterr..

[21] Stabili L., Giangrande A., Arduini D., Borghese J., Petrocelli A., Alabiso G., Ricci P., Cavallo R.A., Acquaviva M.I., Narracci M. (2023). Environmental Quality Improvement of a Mariculture Plant after Its Conversion into a Multi-Trophic System. Sci. Total Environ..

[22] Borghese J., Giangrande A., Arduini D., Trani R., Doria L., Anglano M., Aguilo-Arce J., Toso A., Putignano M., Rizzo L. (2025). Exploring the Potential Effects of IMTA on Water Column Seston through Intensive Short-Time Cycles Approach. Mar. Pollut. Bull..

[23] Giangrande A., Licciano M., Arduini D., Borghese J., Pierri C., Trani R., Longo C., Petrocelli A., Ricci P., Alabiso G. (2022). An Integrated Monitoring Approach to the Evaluation of the Environmental Impact of an Inshore Mariculture Plant (Mar Grande of Taranto, Ionian Sea). Biology.

[24] Arduini D., Borghese J., Gravina M.F., Trani R., Longo C., Pierri C., Giangrande A. (2022). Biofouling Role in Mariculture Environment Restoration: An Example in the Mar Grande of Taranto (Mediterranean Sea). Front. Mar. Sci..

[25] Protection of Animals Used for Scientific purposesText with EEA Relevance (2010). The Directive 2010/63/EU of the European Parliament and of the Council.

[26] Metcalfe J.D., Craig J.F. (2011). Ethical Justification for the Use and Treatment of Fishes in Research: An Update. J. Fish Biol..

[27] (2009). No. 1099/2009 Protection of Animals at the Time of Killing Text with EEA Relevance. In *Council of the European Parliament Council Regulation (EC)*. https://eur-lex.europa.eu/eli/reg/2009/1099/oj/eng.

[28] Tarricone S., Ragni M., Carbonara C., Giannico F., Bozzo F., Petrontino A., Caputi Jambrenghi A., Colonna M.A. (2024). Growth Performance and Flesh Quality of Sea Bass (*Dicentrarchus labrax*) Fed with Diets Containing Olive Oil in Partial Replacement of Fish Oil—With or Without Supplementation with *Rosmarinus officinalis* L. Essential Oil. Animals.

[29] Tarricone S., Caputi Jambrenghi A., Cagnetta P., Ragni M. (2022). Wild and Farmed Sea Bass (*Dicentrarchus labrax*): Comparison of Biometry Traits, Chemical and Fatty Acid Composition of Fillets. Fishes.

[30] Sim Y.J., Cho S.H. (2025). Effect of Partial or Complete Substitution of Fish Meal by Meat Meal in the Feed of Red Sea Bream (*Pagrus Major*) on the Growth Performance and Feed Utilization. Aquac. Nutr..

[31] Tarricone S., Iaffaldano N., Colonna M.A., Giannico F., Selvaggi M., Caputi Jambrenghi A., Cariglia M., Ragni M. (2023). Effects of Dietary Red Grape Extract on the Quality Traits in Juvenile European Sea Bass (*Dicentrarchus labrax* L.). Animals.

[32] Wang Z., Qiao F., Zhang W., Parisi G., Du Z., Zhang M. (2024). The Flesh Texture of Teleost Fish: Characteristics and Interventional Strategies. Rev. Aquac..

[33] Agulheiro-Santos A.C., Machado G., Eusébio T., Lança M.J. (2025). Textural Analysis of Sea Lamprey Muscle From Guadiana and Mondego Rivers (Portugal) Using the Warner-Bratzler Shear Method. J. Texture Stud..

[34] Association of Official Agricultural Chemistry (2000). Official Methods of Analysis of the AOAC.

[35] Folch J., Lees M., Stanley G.H.S. (1957). A simple method for the isolation and purification of total lipides from animal tissues. J. Biol. Chem..

[36] Christie W.W. (2014). Lipid Analysis: Isolation, Separation, Identification and Structural Analysis of Lipids.

[37] Ulbricht T.L.V., Southgate D.A.T. (1991). Coronary Heart Disease: Seven Dietary Factors. Lancet.

[38] Dambrosio A., Quaglia N.C., Colonna M.A., Capuozzo F., Giannico F., Tarricone S., Caputi Jambrenghi A., Ragni M. (2023). Shelf-Life and Quality of Anchovies (*Engraulis encrasicolus*) Refrigerated Using Different Packaging Materials. Fishes.

[39] Martinsdottir E., Schelvis R., Hylding G., Sveinsdottir K. (2009). Fishery Products.

[40] Nguyen M.V., Karnue S., Kakooza D. (2023). Effect of Packaging Method and Storage Temperature on the Sensory Quality and Lipid Stability of Fresh Snakehead Fish (*Channa striata*) Fillets. Food Sci. Technol..

[41] Shewan J.M., Macintosh R.G., Tucker C.G., Ehrenberg A.S.C. (1953). The Development of a Numerical Scoring System for the Sensory Assessment of the Spoilage of Wet White Fish Stored in Ice. J. Sci. Food Agric..

[42] Python The Python Language Reference. https://docs.python.org/3/reference/index.html.

[43] Ghosh A.K., Hasanuzzaman A.F., Islam S.S., Sarower M.G., Mistry S.K., Arafat S.T., Huq K.A. (2025). Integrated Multi-Trophic Aquaculture (IMTA): Enhancing Growth, Production, Immunological Responses, and Environmental Management in Aquaculture. Aquac. Int..

[44] Estévez A., Vasilaki P. (2023). Organic Production of Gilthead Sea Bream (*Sparus aurata*) Using Organic Certified Green Pea Protein and Seaweed. Effects on Growth, Feed Conversion and Final Product Quality. Aquaculture.

[45] Latremouille D.N. (2003). Fin Erosion in Aquaculture and Natural Environments. Rev. Fish. Sci..

[46] Bordignon F., Ferrarese L., Solimeo A., Di Leva V., Trocino A. (2025). Animal-Based Measures for Operational Welfare Indicators at Wholesale Level in Gilthead Seabream (*Sparus aurata*) Reared in the Mediterranean Sea. Aquaculture.

[47] Filipa-Silva A., Monteiro M., Costa R.S., Sá T., Marques A., Valente L.M.P., Figueiredo-Silva C. (2025). Comparative Study of Dietary Selenium Sources on Gilthead Seabream (*Sparus aurata*): Growth, Nutrient Utilization, Stress Response and Final Product Quality. Aquaculture.

[48] Orban E., Sinesio F., Paoletti F. (1997). The Functional Properties of the Proteins, Texture and the Sensory Characteristics of Frozen Sea Bream Fillets (*Sparus aurata*) from Different Farming Systems. LWT-Food Sci. Technol..

[49] Ayala M.D., Santaella M., Martínez C., Periago M.J., Blanco A., Vázquez J.M., Albors O.L. (2011). Muscle Tissue Structure and Flesh Texture in Gilthead Sea Bream, *Sparus aurata* L., Fillets Preserved by Refrigeration and by Vacuum Packaging. LWT-Food Sci. Technol..

[50] Attouchi M., Sadok S. (2012). The Effects of Essential Oils Addition on the Quality of Wild and Farmed Sea Bream (*Sparus aurata*) Stored in Ice. Food Bioprocess Technol..

[51] Sáez M.I., Sabio J., Galafat A., Vizcaíno A.J., Alarcón-López F.J., Moya T.F.M. (2025). Evaluation of White Grape Marc Extract as an Additive to Extend the Shelf-Life of Fish Fillets. Foods.

[52] Abbas K.A., Mohamed A., Jamilah B., Ebrahimian M. (2008). A Review on Correlations between Fish Freshness and pH during Cold Storage. Am. J. Biochem. Biotechnol..

[53] Yildiz M., Şener E., Timur M. (2006). The Effects of Seasons and Different Feeds on Fatty Acid Composition in Fillets of Cultured Gilthead Sea Bream (*Sparus aurata* L.) and European Sea Bass (*Dicentrarchus labrax* L.) in Turkey. Turk. J. Vet. Anim. Sci..

[54] Yildiz M., Şener E., Timur M. (2008). Effects of Differences in Diet and Seasonal Changes on the Fatty Acid Composition in Fillets from Farmed and Wild Sea Bream (*Sparus aurata* L.) and Sea Bass (*Dicentrarchus labrax* L.). Int. J. Food Sci. Technol..

[55] Vasconi M., Caprino F., Bellagamba F., Moretti V.M. (2017). Fatty Acid Composition of Gilthead Sea Bream (*Sparus aurata*) Fillets as Affected by Current Changes in Aquafeed Formulation. Turk. J. Fish. Aquat. Sci..

[56] Meinam M., Deepti M., Madhulika, Ngasotter S., Sundaray J.K., Rather M.A., Ahmad I., Amin A. (2025). Emerging Aquaculture Technologies for Food and Nutritional Security. Food Security, Nutrition and Sustainability Through Aquaculture Technologies.

[57] Calanche J., Pedrós S., Roncalés P., Beltrán J.A. (2020). Design of Predictive Tools to Estimate Freshness Index in Farmed Sea Bream (*Sparus aurata*) Stored in Ice. Foods.

[58] Parlapani F.F., Boziaris I.S., Drosinos E.H. (2024). Detection of Fish Spoilage. Handbook of Seafood and Seafood Products Analysis.

[59] Alasalvar C., Taylor K.D.A., Öksüz A., Garthwaite T., Alexis M.N., Grigorakis K. (2001). Freshness Assessment of Cultured Sea Bream (*Sparus aurata*) by Chemical, Physical and Sensory Methods. Food Chem..

[60] Šimat V., Bogdanović T., Krželj M., Soldo A., Maršić-Lučić J. (2012). Differences in Chemical, Physical and Sensory Properties during Shelf Life Assessment of Wild and Farmed Gilthead Sea Bream (*Sparus aurata* L.). J. Appl. Ichthyol..

[61] Lougovois V.P., Kyranas E.R., Kyrana V.R. (2003). Comparison of Selected Methods of Assessing Freshness Quality and Remaining Storage Life of Iced Gilthead Sea Bream (*Sparus aurata*). Food Res. Int..

