# Supplementary material for: Flesh Quality, Shelf Life, and Freshness Assessment of Sea Bream Reared in a Coastal Mediterranean Integrated Multi-Trophic Aquaculture System
Source: Animals (Basel). 2025 Aug 19;15(16):2425. doi: 10.3390/ani15162425 (PMC12383101; doi:10.3390/ani15162425)
Supplement: Supplementary file 1 [file animals-15-02425-s001.zip › animals-3801551-supplementary.pdf]

Table S1. Freshness assessment of sea bream fillets.

Parameters	Control			IMTA			SEM[1]	Effects[2]		
	1	7	14	1	7	14		R	S	R x S
QIM	2.50^{aY}	7.88^{XY}	17.63^{aX}	1.60^{bY}	7.22^{XY}	14.23^{bX}	0.867	0.038	0.001	0.002
TORRY	9.71^{x}	8.50^{xy}	7.90^{y}	9.96^{x}	8.75^{xy}	7.96^{y}	1.186	0.059	0.039	0.083

[1] Standard error of means; [2] Effects: R, rearing system; S, storage time; R x S, interaction. Differences between rearing systems: a; b: $p < 0.05$; differences between the storage periods: X, Y: $p < 0.01$; x, y: $p < 0.05$.
